# Long-term sheep grazing reduces fungal necromass carbon contribution to soil organic carbon in the desert steppe

**DOI:** 10.3389/fmicb.2024.1478134

**Published:** 2024-10-10

**Authors:** Tianqi Zhao, Naijing Lu, Jianying Guo, Xin Zhang, Jing Liu, Mengli Zhao

**Affiliations:** ^1^Yinshanbeilu Grassland Eco-Hydrology National Observation and Research Station, China Institute of Water Resources and Hydropower Research, Beijing, China; ^2^Key Laboratory of Grassland Resources of the Ministry of Education, Key Laboratory of Forage Cultivation, Processing and High Efficient Utilization of the Ministry of Agriculture and Rural Affairs, Inner Mongolia Key Laboratory of Grassland Management and Utilization, College of Grassland, Resources and Environment, Inner Mongolia Agricultural University, Hohhot, China

**Keywords:** carbon sequestration, grassland ecosystem, grazing intensity, microbial community, stocking rate

## Abstract

Grazing has been shown to impact the soil environment and microbial necromass carbon (MNC), which in turn regulates soil organic carbon (SOC). However, the carbon sequestration potential of fungi and bacteria under different stocking rates remains unclear, limiting our understanding of soil carbon sequestration in grazing management. In 2004, we established grazing experiments in the desert steppe of northern China with four stocking rates. Our findings indicate that MNC decreased under moderate and heavy grazing, while light grazing did not significantly differ from no grazing. Notably, the reduction in fungal necromass carbon, rather than bacterial necromass carbon, was primarily responsible for the decreased contribution of MNC to SOC. This difference is attributed to the varying effects of sheep grazing on fungal and bacterial community characteristics, including richness, diversity, and composition. Thus, to accurately predict carbon dynamics in grassland ecosystems, it is essential to consider that the ecological impacts and carbon sequestration potential of microbial communities may vary with different grazing management practices.

## 1 Introduction

Grasslands play an integral role in the Earth’s terrestrial carbon cycle, functioning as significant carbon sinks (Bardgett et al., 2021). Grazing, as a grassland management practices, induces small changes in soil organic carbon (SOC) that can significantly impact global climate change and food security (Bai and Cotrufo, 2022). Historically, a substantial portion of research on grazing has focused on developing strategies to enhance economic returns (Zhang M. et al., 2023). However, in the context of global climate change and the urgent need for sustainable development (Soergel et al., 2021), the scientific management and regulation of grazing intensity to optimize the carbon sequestration capacity of grasslands has become a pressing and challenging issue (Hao et al., 2024).

Soil microbial necromass, mainly composed of particulate organic matter from microbial cell membrane fragments, is defined by its carbon content, known as microbial necromass carbon (MNC) (Sokol et al., 2022). A growing body of research has demonstrated that microbial necromass is a significant component of soil organic matter (Wang et al., 2021a). Carbon fractions generated by microbial metabolism exist in soils in a very stable form, allowing them to persist in natural settings for extended periods, often lasting years or even decades (Romanowicz et al., 2023). However, it remains unclear whether MNC content and its contribution to SOC are altered when comparing ungrazed soils with those subjected to long-term grazing disturbances and varying stocking rates.

Previous studies have identified three primary factors driving MNC content: plant carbon input, soil physicochemical properties, and microbial communities (Wang et al., 2023). Plant-derived carbon inputs, particularly from root systems, are crucial for stimulating microbial activity and the generation of MNC and SOC (Lange et al., 2015). Plant-derived organic matter undergoes a series of biochemical transformations within the soil, including microbial metabolic activity and extracellular enzyme-catalyzed breakdown, before it can be converted into stable MNC (Angst et al., 2021). Grazing can also alter the microbial habitat by influencing soil physicochemical properties, such as pH, soil bulk density, and aggregate fraction, which in turn affects the accumulation of MNC (Bastida et al., 2021).

The biological characteristic of microbial necromass leads to diverse decomposition rates, as fungi and bacteria differ in the structure of their cell walls and their chemical composition (Gentry et al., 2015). Changes in microbial community composition may influence carbon accumulation in microbial necromass, as evidenced by the considerable differences in decomposition rates between bacterial and fungal communities (Jiao et al., 2020). The principal tenets of the ‘microbial carbon pump’ theory, which have been corroborated by studies on SOC and MNC (Jiao et al., 2024), suggest that substances synthesized by microbial cells are integrated into SOC and stabilized through intricate interactions (Niehaus et al., 2019). Additionally, Yang et al. (2022b) found that enhanced microbial community abundance can improve microbial activity and carbon turnover efficiency, thereby accelerating MNC accumulation. A substantial body of evidence suggests that MNC and its components exhibit considerable heterogeneity across diverse habitats (Wang et al., 2021a). Therefore, understanding the formation of MNC fractions and characterizing microbial community structures are essential steps toward comprehending MNC accumulation in ecosystems (Wang et al., 2021b).

Based on these findings, this study hypothesizes that grazing could reduces MNC content and its contribution to SOC, with this reduction increasing as stocking rates (H1). Additionally, it is hypothesized that grazing regulates the reduction of fungal and bacterial necromass carbon by altering plant carbon inputs, the soil environment, and microbial community structure (H2). In carbon cycle models, clarifying the relationship between indicators such as MNC and SOC is crucial for evaluating the capacity of soil carbon sequestration under grazing conditions (Crowther et al., 2015; Buckeridge et al., 2022). Understanding these mechanisms will lead to a better understanding of carbon sequestration processes under long-term grazing.

## 2 Materials and methods

### 2.1 Study site and experimental design

The study site was located in the desert steppe of Siziwang Banner, Inner Mongolia, China (41°46′44″N, 111°53′42″E, elevation 1,456 m, Supplementary Figure S1). The region experiences a semi-arid climate, characterized by an annual mean temperature of 3.7°C and an annual mean precipitation of 228 mm (Zhang B. et al., 2023). Most precipitation occurs during the summer months, which are marked by high temperatures and low humidity, while spring is typically dry with strong winds. The dominant vegetation in the area includes *Stipa breviflora*, *Cleistogenes songorica*, and *Artemisia frigida*. The soil at the study site is classified as sandy loam and is categorized as Kastanozem according to the Food and Agriculture Organization classification system (Zhao et al., 2024).

In June 2004, a randomized complete block design with three replicates was implemented for a sheep grazing management experiment across 52.8 hectares (ha) of pasture. The experiment was arranged in 12 plots, each measuring 4.4 ha (Supplementary Figure S1). Four different stocking rates were tested: 0, 0.15, 0.30, and 0.45 sheep hectare ha^−1^ month^−1^. The corresponding treatments were: (i) no grazing (NG); (ii) light grazing (LG) with 4 sheep per plot; (iii) moderate grazing (MG) with 8 sheep per plot; and (iv) heavy grazing (HG) with 12 sheep per plot. During the grazing period from June to November, sheep were allowed to graze freely from 6:00 to 18:00 and were relocated to enclosed pens at night.

### 2.2 Sampling and measurements

In July 2021, plant species within three 1-m^2^ quadrats were mowed to ground level. For each quadrant, three soil samples were taken to assess root biomass at a depth of 0–10 cm. After carefully washing the soil from the roots, they were dried in an oven at 65°C for 48 h. Plant carbon inputs (grams (g) C m^−2^) from the shoots and roots were calculated using Equation 1 as follows (Liu et al., 2021):


(1) ΔLc=Mx×Cx

The variable *Mx* represents the mass of shoots and roots (in grams per square meter), while *Cx* denotes the carbon content (in grams of carbon per gram of dry matter) of the shoots and roots. The term *∆Lc* signifies the carbon input (in grams of carbon per square meter) from the shoots and roots. The carbon content of the shoots and roots was determined using a C/N elemental analyzer (Multi-N/C 2100, Analytik Jena AG, Jena, Germany).

After the plant samples were collected, soil samples were taken from the 0–10 cm layer within each quadrat. A 7.5 cm diameter soil corer was used to collect samples diagonally between the corners of each quadrant. Five soil cores were taken from each quadrat and combined into a single sample. Additionally, a 100cm^3^ stainless steel ring was used to obtain soil samples for calculating soil bulk density and capillary water capacity. The 36 soil core samples were stored on ice for 24 h before being transported to the laboratory, where they were sieved through a 2-mm mesh to remove stones and root fragments. A 100-grams portion of each soil sample was stored at −80°C for DNA extraction. The remaining soil samples were air-dried and subsequently analyzed for their physicochemical properties.

### 2.3 Soil physicochemical properties

Soil pH was measured using a pH electrode (Analytik Jena, Jena, Germany) at a fresh soil-to-water ratio of 1:2.5. The air-dried soils samples were then analyzed for total carbon content using an elemental analyzer (Shimadzu Corp., Kyoto, Japan), following grinding in a ball mill (MAT-253, Thermo Fisher Scientific, USA). Soil organic carbon was calculated by subtracting inorganic carbon from total carbon, with inorganic carbon being determined through volumetric analysis using the hydrochloric acid (HCl) reaction, as previously described by Zhang et al. (2020). The capillary water holding capacity was determined following the methods described by Zhang et al. (2006), and outlined in Equation 2 and subsequent steps. Initially, water was applied to the soil within a stainless steel ring for 2 h. The samples were then weighed (*m_1_*) after being left on dry sand for 48 h to allow complete drainage of water. Finally, the soil was removed from the ring, placed in an aluminum box, and dried to a constant weight (*m_0_*).


(2) $$\mathrm{Capillary water holding capacity}\;\left(\%\right)=\left(m_{1}–m_{0}\right)/m_{0}\times 100$$

To collect the soil aggregates *in situ*, a pit was excavated in each quadrant to a depth of 0–10 cm. The soil samples were placed in aluminum boxes and allowed to air dry in the laboratory. The wet-sieving procedure outlined by Elliott (1986) was then used to separate the soil samples into three aggregate-size fractions: large aggregates (>0.25 mm), small aggregates (0.053–0.25 mm), and microaggregates (<0.053 mm).

### 2.4 Microbial biomass carbon and MNC

Microbial biomass carbon was determined using a fumigation extraction method with chloroform (Oren et al., 2018). After fumigation, soils (fumigated and non-fumigated) were extracted with 0.5 M K_2_SO_4_, and the dissolved organic carbon (DOC) concentrations were measured using a C/N analyzer (Analytik Jena, Jena, Germany). The difference in DOC between the fumigated and non-fumigated soils was used to calculate microbial biomass carbon, applying a conversion coefficient of 0.45.

To determine MNC, the soil’s amino sugar carbon content was analyzed (Zhang and Amelung, 1996). Amino sugars, including glucosamine (GluN), galactosamine (GalN), mannosamine (ManN), and muramic acid (MurA), were identified and quantified following the methodology of Zhang and Amelung (1996). Soil samples were hydrolyzed with 10 milliliters of 6 M HCl for 8 h, after which the solution was freeze-dried. Methanol was added to the freeze-dried supernatants, and the residues were centrifuged to extract the amino sugars. These sugars were than reacted with acetic anhydride, 4-(dimethylamino) pyridine, and hydroxylamine hydrochloride to form derivatives. The resulting amino sugars derivatives were separated on a regular polysiloxane DB-5MS column using an Agilent 6,890 gas chromatograph (Agilent Technologies, Wilmington, DE, USA). The derivatives were identified by comparing their retention times to internal standards containing GluN, GalN, ManN, and MurA. Fungal necromass carbon was calculated using Equation 3, and bacterial necromass carbon was calculated using Equation 4, as follows (Li Y. Z. et al., 2024).


(3) $$\mathrm{Fungal necromass carbom}=\left(\frac{\mathrm{GluN}}{179.17}-2\times \frac{\mathrm{MurA}}{251.23}\right)\times 179.17\times 9$$

(4) Bacterial necromass carbom=MurA×45

The molecular weights of GluN and MurA are 179.17 and 251.23, respectively. Their conversion coefficients to fungal necromass carbon and bacterial necromass carbon are 9 and 45, respectively. The sum of fungal and bacterial necromass carbon represents the total microbial necromass carbon (Liu et al., 2024).

### 2.5 Soil extracellular enzyme assays

The hydrolytic enzymes *α*-1,4-glucosidase, *β*-1,4-glucosidase, and β-1,4-N-acetylglucosaminidase present in fresh soil samples were evaluated fluorometrically using methylumbelliferone-labeled substrates (Mori et al., 2021). Briefly, 1 g of dry soil was combined with 50 mL of 50-mM acetate buffer (pH 5.0) in a 100-mL centrifuge tube using a Polytron homogenizer. The mixture was then transferred to a round, wide-mouthed beaker. The centrifuge tube was rinsed, and an additional 50 mL of acetate buffer was added to the beaker containing the soil suspension. Next, the soil suspension, along with the buffer, 10-μM references standards, and 200-μM substrates, was pipetted into the wells of a black 96-well microplate. Fluorescence was measured using a microplate fluorometer equipped with 365-nanometer (nm) excitation and 450-nm emission filters.

### 2.6 DNA extraction and amplicon generation

DNA was extracted from each of the 226 soil samples using the Fast DNA^®^ SPIN Kit for Soil (MP Biomedicals). The concentration and purity of the extracted DNA were assessed using a NanoDrop One spectrophotometer (Thermo Fisher Scientific, Massachusetts, USA). Specific primer sets were used to amplify the 16S rRNA and its genes in two distinct regions: bacterial 16S (V3-V4, primers 338F, and 806R) and fungal 18S (ITS2, primers ITS5-1737F and ITS2-2043R), with a 12-base pair (bp) barcode incorporated for identification (Gong et al., 2021). These primers were synthesized according to the specifications provided by Invitrogen (Invitrogen, Carlsbad, CA, USA). The PCR reaction mixture contained 50 microliters of 2x Premix Taq (Guangdong Magigene Biotechnology Co. Ltd., Guangzhou, China), 1 μL of each primer (forward and reverse, 10 μM), and 3 μL of DNA template (20 ng/μL). The amplification process was carried out as follows: an initial denaturation step at 94°C for 5 min, followed by 30 cycles of 30 s at 94°C for denaturation, 30 s for annealing, 30 s for extension, and a final elongation step of 30 s at 72°C. The Bio-Rad S1000 thermocycler (Bio-Rad Laboratory, CA, USA) was used for the PCR process.

### 2.7 High-throughput sequencing

The NEBNext^®^ UltraTM II DNA Library Prep Kit for Illumina^®^ (New England Biolabs, MA, USA) was used to construct sequencing libraries, with library quality assessed using a Qubit@ 2.0 Fluorometer (Thermo Fisher Scientific, MA, USA.) Amplicon libries were sequenced on an Illumina Nova6000 platform (Guangdong Magigene Biotechnology Co. Ltd., Guangzhou, China) using 250 bp paired-end reads. To ensure high-quality raw data, a sliding window approach (-W 4 -M 20) was applied, and *Fastp* (version 0.14.1)^1^ was selected for data filtering. Primers were removed from the raw reads using *cutadapt*,^2^ which eliminates primer information from both ends of the sequences, resulting in clean paired-end reads. The UCHIME algorithm was then used to detect and remove chimeric sequences, producing the final set of clean reads. For taxonomic classification, soil bacteria amplicon sequences were compared against the Silva database,^3^ while fungal reads were aligned with the Unite database.^4^ Sequences with >97% similarity were grouped into the same operational taxonomic unit (OTU) using *UPARSE* (version 7.0.1001).^5^ OTUs with fewer than three reads, which could result from sequencing artifacts, were excluded from further analysis. The representative sequence for each OTU was then identified. To determine the taxonomic classification of bacterial and fungal communities, the Silva and Unite databases were analyzed using Mothur v. 1.48.0 and the National Center for Biotechnology Information’s Basic Local Alignment Search Tool (NCBI BLAST) algorithms. OTU abundance data were normalized to the sample with the fewest sequences, enabling accurate determination of the bacterial and fungal community compositions across samples.

### 2.8 Statistical analysis and modeling

A mixed-effects model was applied using the *lme4* package to assess the influence of different stocking rates on MNC, soil environments, plant carbon inputs, and microbial community indices. In this model, stocking rates were treated as fixed effects, while blocks were treated as random effects. Multiple comparisons were performed using an LSD test, with a significance threshold set at *p <* 0.05. Regression analysis was utilized to evaluate the relationships between plant carbon input, soil physicochemical properties, MNC fractions, and microbial community indices. All statistical analyses were executed in the R software environment, version 4.1.2.^6^

The study aimed to determine the relative impacts of plant carbon input, microbial community structure, microbial biomass carbon, and soil environmental factors on MNC under varying stocking rates. This analysis was conducted using structural equation modeling (SEM), with the initial model illustrated in Supplementary Figure S2. Before performing SEM, data processing was undertaken as follows: a multivariate function index was constructed for each group using principal component analysis (PCA), which included tightly correlated variables representing plant carbon input, soil environment, and microbial community structure. Only factors significantly correlated with soil organic carbon (SOC) were retained in the PCA for each group. In the subsequent SEM analysis, the first principal component was integrated as a new variable. Model fit was evaluated using Chi-square tests, *p*-values, comparative fit indices, AIC values, and the root mean square error of approximation. Structural equation modeling analysis was carried out using the AMOS software, version 2.0 (AMOS Development Corporation, Chicago, IL, USA).

## 3 Results

### 3.1 Effect of different stocking rates on fungal and bacterial necromass carbon and soil organic carbon

**Figure 1:**
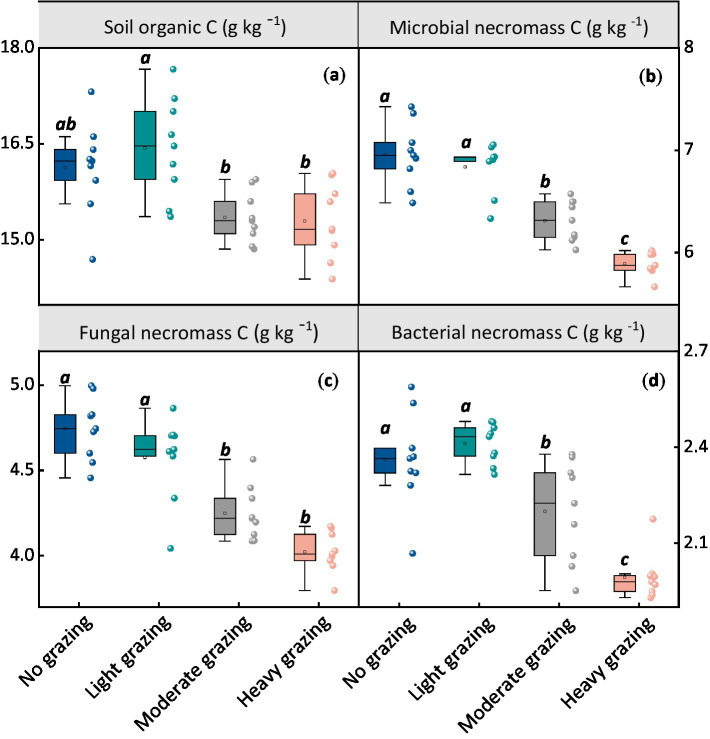
Effects of different stocking rates on soil organic carbon (SOC) and microbial necromass carbon content. Each panel represents **(a)** SOC content, **(b)** total microbial necromass carbon content, **(c)** fungal necromass carbon content, and **(d)** bacterial necromass carbon content at varying stocking rates. Different lowercase letters represent significant differences among grazing treatments (*p* < 0.05). The box boundaries represent the 25th and 75th percentiles, while the black lines indicate the means (averages).

After 17 years of grazing, SOC levels were significantly lower under both moderate and heavy grazing compared to no grazing. Specifically, SOC was approximately 4% lower in moderate grazing (15.4 g/kg) and heavy grazing (15.3 g/kg) compared to no grazing (16.1 g/kg), while light grazing (16.4 g/kg) showed no significant difference from no grazing (Figure 1). Similarly, microbial necromass carbon (MNC), which includes fungal necromass carbon and bacterial necromass carbon, exhibited a comparable trend. MNC ranged from 5.89 to 6.96 g/kg, with fungal necromass carbon ranging from 4.02 to 4.74 g/kg and bacterial necromass carbon from 1.87 to 2.26 g/kg. All these indicators decreased significantly with increasing stocking rates (*p <* 0.05, Figure 1).

**Figure 2:**
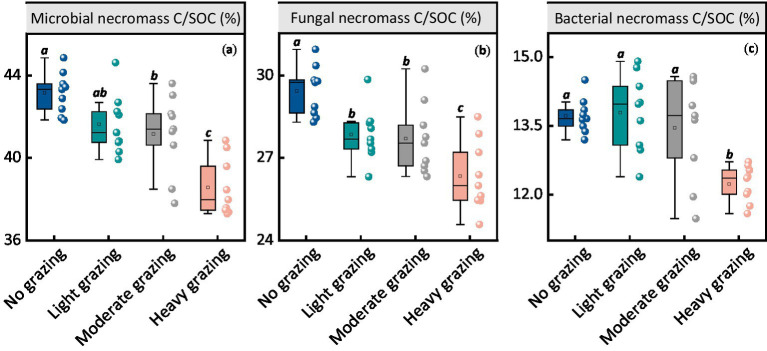
Ratios and contents of microbial necromass carbon (MNC) and its components at different stocking rates. Each panel represents the ratios of **(a)** microbial necromass carbon to SOC, **(b)** fungal necromass carbon to SOC, and **(c)** bacterial necromass carbon to SOC at different stocking rates. Different lowercase letters represent significant differences among grazing treatments (*p* < 0.05). The box boundaries represent the 25th and 75th percentiles, while the black lines indicate the means (averages).

The contributions of MNC to SOC was notably affected by grazing intensity (Figure 2a). The proportion of MNC contributing to SOC decreased with higher stocking rates. For no grazing, MNC contributed 43.2% to SOC, which decreased to 41.6% under light grazing, 41.2% under moderate grazing, and 38.6% under heavy grazing (*p <* 0.05, Figure 2a). The pattern for fungal necromass carbon contributions to SOC and total MNC was similar, showing a significant decrease under higher stocking rates (*p <* 0.05, Figure 2b). Additionally, the contribution of bacterial necromass carbon to SOC was significantly reduced under heavy grazing conditions (*p <* 0.05, Figure 2c).

### 3.2 Response of fungal and bacterial necromass carbon to abiotic and biotic factors

**Figure 3:**
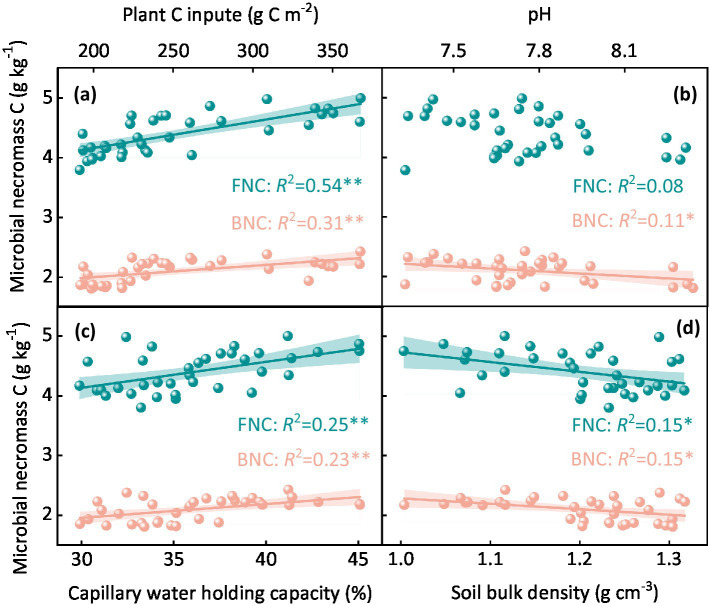
Correlation between fungal and bacterial necromass carbon and various soil and plant factors including **(a)** plant carbon input, **(b)** soil pH, **(c)** capillary water holding capacity, and **(d)** bulk density. The *R*^2^ values indicate the strength of the correlation, with statistical significance assessed at *p* < 0.05.

A significant increase in MNC was observed with increasing plant carbon inputs (*p* < 0.05, Figure 3; Supplementary Figure S3). Both fungal and bacterial necromass carbon showed positive correlations with plant carbon input, with fungal necromass carbon (*R*^2^ = 0.54, *p* < 0.05) and bacterial necromass carbon (*R*^2^ = 0.31, *p* < 0.05) increasing as plant carbon inputs increased (Figure 3a; Supplementary Figure S3). The impacts of different sheep stocking rates on soil physicochemical properties were also significant (*p* < 0.01, Supplementary Table S1; Supplementary Figure S3). Fungal necromass carbon did not show a significant correlation with soil pH (*p* > 0.05, Figure 3b). However, bacterial necromass carbon decreased significantly with increasing soil pH (*R*^2^ = 0.11, *p* < 0.05, Figure 3b). Both fungal and bacterial necromass carbon were significantly positively correlated with soil capillary water holding capacity (*R*^2^ = 0.25 and 0.23, *p* < 0.05, respectively, Figure 3c). Conversely, the MNC decreased significantly with increasing soil bulk density (*R*^2^ = 0.15, *p* < 0.05, Figure 3d).

**Figure 4:**
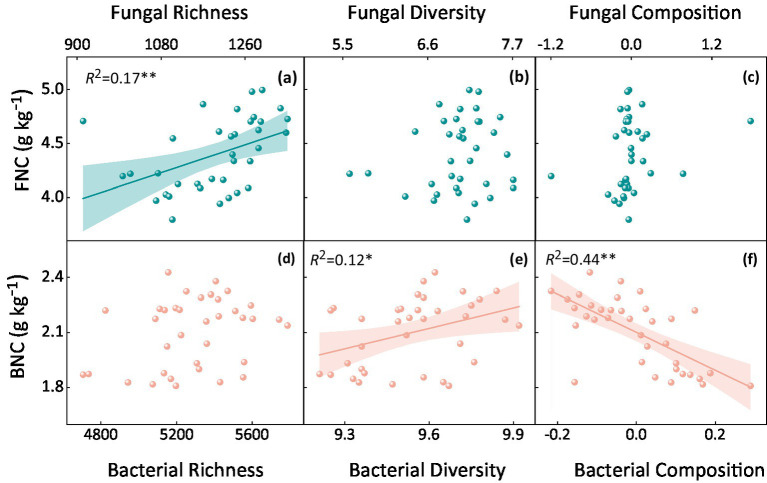
Correlation between fungal and bacterial necromass carbon and their **(a, d)** richness, **(b, e)** diversity, and **(c, f)** composition. FNC denotes fungal necromass carbon, and BNC denotes bacterial necromass carbon. The *R*^2^ values indicate the strength of the correlation, with statistical significance assessed at *p* < 0.05.

Soil microbial factors, including community richness, diversity, and composition, decreased significantly with increasing stocking rate (*p <* 0.05, Supplementary Table S1). There was a significant positive correlation between fungal richness, bacterial diversity, and MNC (*p <* 0.01, Figure 4). Additionally, a strong correlation was found between MNC and certain dominant bacteria (Supplementary Figure S3). Specifically, MNC was negatively correlated with Actinobacteria and positively correlated with Proteobacteria (*p <* 0.05, Supplementary Figure S3).

### 3.3 Mechanisms governing fungal and bacterial necromass carbon

**Figure 5:**
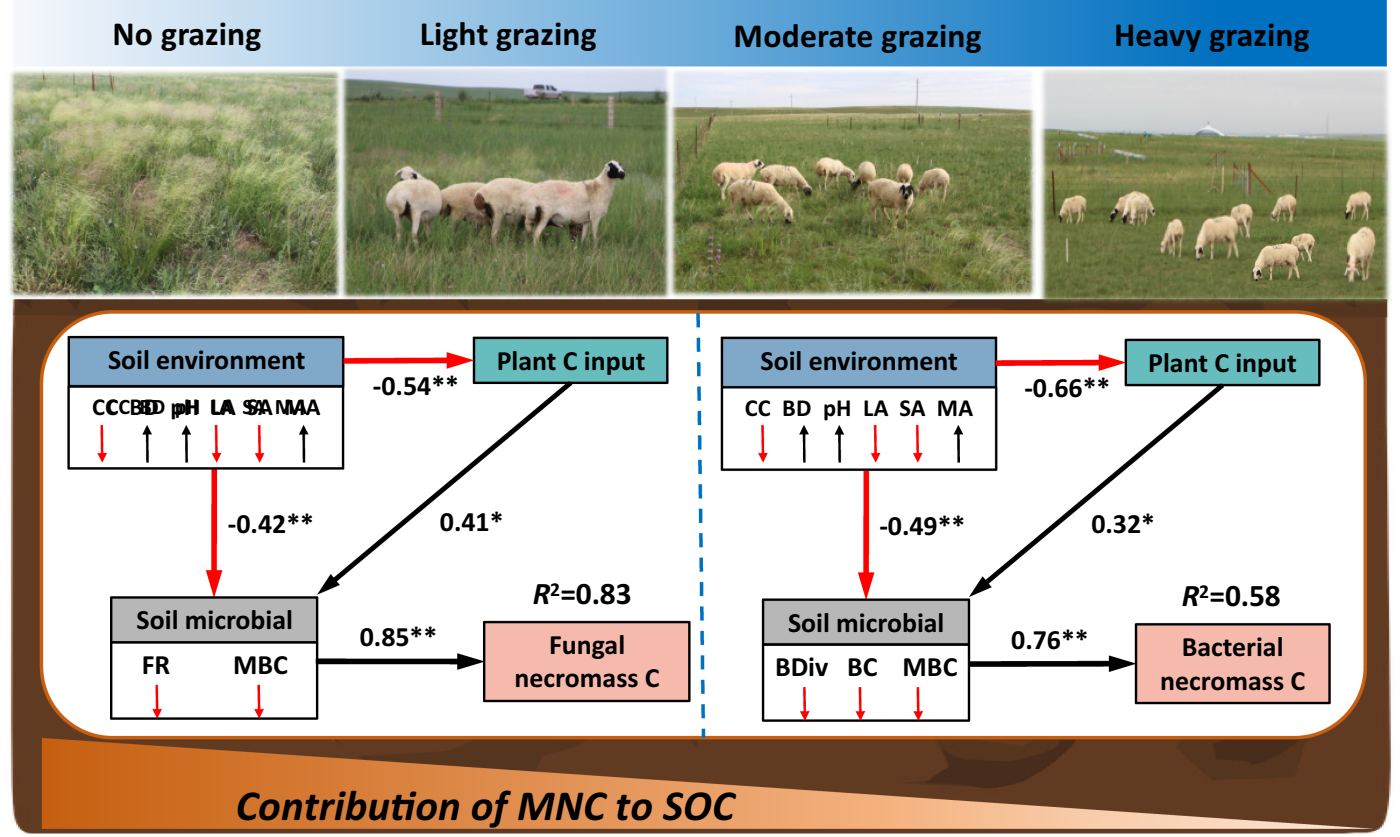
Structural equation model (SEM) illustrating the effects of grazing on fungal and bacterial necromass carbon. Boxes represent the measured variables within the model. Values adjacent to arrows indicate standardized path coefficients, reflection the degree of effect of each relationship. Black lines denote significant positive correlations, while red lines represent significant negative correlations. Variables include: capillary water holding capacity (CC), bulk density (BD), microbial biomass carbon (MBC), fungal richness (FR), bacterial diversity (BDiv), bacterial composition, LA (large aggregates), SA (small aggregates), and MA (microaggregates). Significance levels are denoted as *p < 0.05, **p < 0.01, ***p < 0.001.

SEM models revealed that the combined effects of plant carbon input, soil environment, and soil microbes significantly influenced fungal and bacterial necromass carbon under long-term grazing in the desert steppe (Figure 5; Supplementary Table S2). As stocking rates increased, plant carbon input decreased, while soil environmental factors such as, capillary water holding capacity and large aggregate fractions also declined. Concurrently, bulk density and microaggregate fractions increased. These changes directly led to a reduction in the richness of fungal and microbial biomass carbon, which in turn, caused a decrease in fungal necromass carbon (*R*^2^ = 0.83, Figure 5). For bacterial necromass carbon, increased stocking rates were associated with lower soil pH and higher small aggregate fractions. These changes reduced the diversity and composition of bacterial and microbial biomass carbon, resulting in a decrease in bacterial necromass carbon (*R*^2^ = 0.58, Figure 5). Interestingly, microbial factors had a strong positive influence on necromass carbon, with path coefficients of 0.85 for fungal necromass carbon and 0.76 for bacterial necromass carbon, indicating their significant regulatory role (Figure 5).

## 4 Discussion

### 4.1 Grazing reduces MNC and its contribution to SOC

Our findings align with previous studies and conceptual models that have demonstrated grazing’s risk of reducing microbial necromass carbon (MNC) and soil organic carbon (SOC) (Wang et al., 2021a; Ni et al., 2021; Buckeridge et al., 2022). The study observed a significant decrease in the MNC to SOC ratio, indicating that grazing led to a faster reduction in MNC compared to SOC. Specifically, sheep grazing considerably reduces MNC’s contribution to SOC, with this effect becoming more pronounced at higher stocking rates. Despite a 4% reduction in SOC content over 17 years of intensive grazing, the desert steppe’s low soil organic matter content means that short-term disturbances have minimal impact on SOC levels (Zhuo et al., 2022). Additionally, the presence of sheep dung and undecomposed plant residues from grazing had minimal effect on SOC variation (Zhu et al., 2020). The study indicates a potential 15% reduction in MNC due to grazing. This decline in MNC may be attributed to reduced microbial carbon pumping efficiency caused by changes in plant carbon supply, soil physicochemical characteristics, and microbial populations following grazing (Qian et al., 2023).

Root deposition carbon is represents the primary source of organic carbon in grassland soils. The sharp decline in plant carbon input due to increased grazing intensity significantly reduces microbial residue production efficiency (Shen et al., 2020). Grazing can also decrease microbial turnover and activity, leading to a delayed accumulation of MNC (Bai and Cotrufo, 2022). According to the microbial carbon pump theory, microbial enzymes modify some of the newly added organic matter in the soil (Yang et al., 2024). Our investigation found a reduction in the activities of three carbon-cycling-related enzymes as stocking rates increased (Supplementary Table S1). These findings are consistent with Yang et al. (2022a), who observed a positive correlation between plant carbon input and soil enzyme activity (Supplementary Figure S3). Therefore, grazing supports our first hypothesis by reducing plant carbon input, which subsequently lowers enzyme activity and potentially limits MNC accumulation (Roy and Bagchi, 2022).

It is reasonable to conclude that grazing can significantly alter soil physicochemical characteristics. Trampling increases bulk density and compaction, leading to reduced habitat suitability for microbes due to limited oxygen and water availability (Pan et al., 2021). Higher stocking rates further degrade the microbial survival environment, resulting in decreased efficiency of microbial necromass accumulation (Han et al., 2024). This degradation negatively impacts the accumulation of new microbial biomass carbon (Mason-Jones et al., 2022). Furthermore, increased stocking rates lead to fragmentation of soil aggregates, releasing more carbon into the soil for decomposition (Mustafa et al., 2020). Consequently, the production of microbial necromass is constrained by the reduction in microbially-derived carbon (Hou et al., 2024). Our preliminary hypothesis is supported by these findings, as the process of microbial necromass reduction is exacerbated with higher stocking rates.

Our research shows that grazing has led to significant changes in the richness, diversity, and composition of bacterial and fungal communities, which are closely linked to overall MNC content. This effect is attributed to the reduction in root carbon deposition caused by grazing, which decreases microbial abundance and their resilience to environmental changes (Zeng et al., 2024). As a result, there is a reduced production of microbial residues (Yang et al., 2020). Grazing has been found to simplify microbial community structure, negatively impacting microbial turnover and biomass accumulation, as noted by Wilson et al. (2018). Furthermore, Wang et al. (2021a) highlighted that soil microbial diversity is a crucial indicator of soil organic carbon (SOC) formation in a global context. Consequently, our findings align with previous research, reinforcing the positive correlation between MNC and microbial richness.

### 4.2 Grazing affects SOC mainly through fungal necromass carbon rather than bacterial necromass carbon

Previous literature, including field studies and meta-analyses, has shown that in global grassland ecosystems, MNC tends to increase alongside SOC content (Beillouin et al., 2023). Our current study revealed that fungal necromass carbon exhibited variation in response to stocking rates, contributing a greater proportion to SOC compared to bacterial necromass carbon. Two potential explanations are proposed for this phenomenon. First, fungal organisms contribute more to SOC than bacterial organisms (27.8% vs. 13.3%, respectively). Second, fungal necromass is more sensitive to grazing disturbances compared to bacterial necromass. Our findings indicate that while bacterial necromass carbon was significantly reduced under heavy grazing conditions, fungal necromass carbon contributed less to SOC across all stocking rates. This outcome may be attributed to the inherent differences between bacteria and fungi: fungal species richness is positively correlated with grazing, whereas bacterial richness and MNC are negatively affected. Grazing impacts fungi more severely because they function predominantly in the mycelial state, whereas bacteria exist primarily as single cells (Lekberg et al., 2021). According to Yang et al. (2022b), fungi play a crucial role in breaking down complex organic matter in soils and produce carbohydrate-active hydrolases and oxidases that accelerate the turnover of necromass and live microbial biomass (Yan et al., 2020). Thus, high stocking rates in grazing management can significantly disrupt microbial community structure and functions, ultimately impairing carbon sequestration, as evidenced by the decreased contributions of both bacterial and fungal necromass carbon to SOC (Savian et al., 2018).

Sheep excreta and trampling have been shown to significantly alter the soil environment, impacting factors such as soil capillary water holding capacity, bulk density, and pH, as well as the structure of grassland soils, including aggregate fractions (Mosier et al., 2021; Reinhart et al., 2021). A study in semiarid grasslands found that elevated soil moisture can enhance the accumulation of microbial residues (Li Y. et al., 2024). Additionally, Ilek et al. (2015) identified a strong correlation between soil capillary water holding capacity and organic matter. Similarly, a robust positive correlation was observed between fungal and bacterial necromass carbon and soil capillary water retention capacity (Bickel and Or, 2020). In arid and semi-arid grasslands, soil water is the primary environmental factor limiting soil microbial activity, especially during the growth season (Huang et al., 2019). In non-grazed and lightly grazed soils with adequate water holding capacity, the energy and nutrients from organic matter decomposition support microbial growth and reproduction, promoting the accumulation of microbial necromass (Hayer et al., 2022). Conversely, moderate and heavy grazing leads to increased soil bulk density and reduced water-holding capacity due to trampling, resulting in poorer soil conditions and reduced microbial necromass contribution to SOC (Zhao et al., 2024). Our study revealed a negative correlation between soil pH and bacterial necromass carbon, with higher pH levels observed in soils under increased stocking rates. Previous research has shown that soil pH is a key factor influencing the structure of soil microbial communities, particularly bacterial (Kang et al., 2021). Our findings also demonstrate a significant correlation between the relative abundance of Proteobacteria, the dominant soil bacteria, and soil pH, MNC, and SOC (Supplementary Figure S3).

The long-term accumulation of SOC from microbial activity is physically protected by soil microaggregate fractions (Bronick and Lal, 2005; Ma et al., 2018; Wang et al., 2021a). Our research found that while soil microaggregates increased with higher stocking rates, there was a negative correlation between microaggregate fractions and MNC (Supplementary Figure S3). In contrast, MNC was positively correlated with both large and small soil aggregates. This can be attributed to the increased soil bulk density and the protective role of microaggregates in preserving organic matter due to heavy grazing and intense livestock trampling (Saini, 1966; Stavi et al., 2008; Wu et al., 2012). Heavy grazing and trampling constrain water availability and fine root growth (Schmitz et al., 2018), which in turn diminishes microbial activity (Buckeridge et al., 2022; Ni et al., 2020). Previous studies suggest that improved grazing management practices, such as rotational grazing and reduced stocking rates, can enhance microbial necromass accumulation and carbon sequestration in temperate grasslands (Buckeridge et al., 2020; Hou et al., 2024; Lauer et al., 2011; Wang et al., 2024). Therefore, effective grazing management is crucial when evaluating soil carbon sequestration and running model simulations. The evidence underscores that implementing sound grazing practices can promote the accumulation of microbial necromass and SOC in pasture ecosystems (Peng et al., 2024). Understanding the complex process of soil organic carbon accumulation requires considering a network of interacting mechanisms (Lehmann et al., 2020). Empirical evidence from long-term disturbances provides valuable insights into these underlying processes.

## 5 Conclusion

Based on our long-term grazing experiment, we found that an appropriate stocking rate (light grazing: 0.15 sheep ha^−1^ month^−1^) effectively maintained soil organic carbon (SOC). Our results indicated that fungal necromass carbon (C) had a greater impact on SOC than bacterial necromass C, due to differing responses of microbial communities to sheep grazing. Increased grazing intensity led to reduced plant carbon input, deterioration of soil conditions, and disruption of soil structure, all of which were significant factors in the decreased formation of microbial residues and their contribution to organic carbon. In conclusion, our research highlights the importance of considering the ecological impacts and potential for carbon sequestration of microbial communities when predicting carbon dynamics in grassland ecosystems. Different grazing regimes can significantly alter these dynamics, emphasizing the need for tailored management strategies to optimize carbon sequestration.

## Data Availability

The original contributions presented in the study are included in the article/Supplementary material, further inquiries can be directed to the corresponding authors.

## Footnotes

^1^https://github.com/OpenGene/fastp

^2^https://github.com/marcelm/cutadapt/

^3^http://www.arb-silva.de/

^4^https://unite.ut.ee/

^5^http://www.drive5.com/uparse/

^6^https://www.r-project.org/
