# Supplementary material for: Long-term sheep grazing reduces fungal necromass carbon contribution to soil organic carbon in the desert steppe
Source: Front Microbiol. 2024 Oct 10;15:1478134. doi: 10.3389/fmicb.2024.1478134 (PMC11499111; doi:10.3389/fmicb.2024.1478134)

Table S1. Effects of different grazing intensities on carbon input of shoots and roots, physicochemical properties, microbial community and enzyme activity (means ± SE). No grazing (no sheep per grazing plot), light grazing (4 sheep per grazing plot), moderate grazing (8 sheep per grazing plot, and heavy grazing (12 sheep per grazing plot). Different lowercase letters within a row represent significant differences for each variable under different grazing treatments.

		No grazing	Light grazing	Moderate grazing	Heavy grazing	*P*-value
Carbon input of shoots and roots	Plant carbon input(g m^{-2})	**341±11.5^{a}**	253±8.9^{b}	221±3.8^{c}	201±3.6^{c}	$P < 0.01$
	Shoot carbon input(g m^{-2})	38.6±1.27^{ab}	**41.3±1.91^{a}**	35.2±1.86^{bc}	33.3±0.92^{c}	$P = 0.03$
	Root carbon input(g m^{-2})	**302±10.72^{a}**	211±10.69^{b}	185±5.45^{b}	168±4.42^{c}	$P < 0.01$
Soil physicochemical properties	pH	7.59±0.02^{b}	7.68±0.14^{b}	**7.87±0.09**^{a}	**7.89±0.21**^{a}	$P = 0.02$
	Soil bulk density(g·cm^{-3})	1.15±0.05^{b}	1.16±0.01^{b}	**1.25±0.01**^{a}	**1.25±0.01**^{a}	$P = 0.02$
	Capillary water holding capacity(%)	**38.32±2.14**^{a}	**39.17±0.09**^{a}	33.91±1.68^{b}	33.62±1.18^{b}	$P = 0.02$
	Large aggregates(%)	**57.45±0.64**^{a}	**58.65±0.63**^{a}	56.27±0.8^{a}	54.1±0.97^{a}	$P < 0.01$
	Small aggregates(%)	**33.94±0.60**^{a}	**32.97±0.60**^{a}	31.5±0.73^{b}	31.2±1.02^{b}	$P = 0.03$
	Microaggregates(%)	8.38±1.34^{c}	8.63±0.12^{c}	12.23±0.25^{b}	**14.7±0.77**^{a}	$P < 0.01$
Soil microbial properties	Microbial biomass carbon(mg·kg^{-1})	**305±12.94^{a}**	**288.31±8.49^{a}**	239.26±5^{b}	206.98±1.63^{c}	$P < 0.01$
	Bacterial richness	**5478±53.2^{a}**	**5381±60.0^{a}**	5201±66.7^{b}	5097±65.3^{b}	$P < 0.01$
	Fungal richness	**1280±30.3^{a}**	**1206±52.5^{a}**	1156±23.6^{b}	1145±16.7^{b}	$P = 0.04$
	Bacterial diversity	**9.66±0.04^{a}**	**9.65±0.06^{a}**	9.42±0.04^{b}	9.43±0.06^{b}	$P < 0.01$
	Fungal diversity	7.21±0.06^{a}	6.96±0.09^{a}	6.88±0.24^{a}	6.98±0.14^{a}	$P > 0.05$
	Bacterial composition	-0.07±0.02^{c}	-0.12±0.03^{c}	0.04±0.03^{b}	**0.14±0.03^{a}**	$P < 0.01$
	Fungal composition	-0.03±0.04^{a}	0.23±0.25^{a}	-0.06±0.22^{a}	-0.13±0.03^{a}	$P = 0.49$
	Actinobacteria(%)	0.27±0.01^{b}	0.21±0.01^{c}	0.26±0.01^{b}	**0.29±0.01^{a}**	$P < 0.01$
	Acidobacteria(%)	0.22±0.01^{c}	**0.28±0.02^{a}**	0.26±0.01^{ab}	0.24±0.01^{bc}	$P < 0.01$
	Proteobacteria(%)	**0.17±0.01^{a}**	**0.17±0.01^{a}**	0.15±0.00^{b}	0.14±0.01^{b}	$P < 0.01$
	Ascomycota(%)	0.36±0.02^{a}	0.37±0.02^{a}	0.37±0.04^{a}	0.38±0.03^{a}	$P > 0.05$
	Basidiomycota(%)	0.19±0.04^{a}	0.11±0.02^{a}	0.17±0.05^{a}	0.20±0.04^{a}	$P > 0.05$
Soil enzyme activities	α-1,4-glucosidase(nmol·g^{-1}·h^{-1})	**2.78±0.18^{a}**	2.04±0.06^{b}	1.75±0.05^{c}	1.39±0.01^{d}	$P < 0.01$
	β-1,4-glucosidase(nmol·g^{-1}·h^{-1})	**26.7±0.45^{a}**	24.7±0.40^{b}	23.6±0.27^{c}	22.2±0.24^{d}	$P < 0.01$
	β-1,4-N-acetylglucosaminidase(nmol·g·g^{-1}·h^{-1})	**6.2±0.40^{a}**	5.1±0.39^{b}	4.32±0.06^{c}	3.6±0.06^{d}	$P < 0.01$

Table S2. Structural equation model parameters.

	χ^2	*P*-value	CFI	RMSEA	AIC
Fungal necromass carbon	3.82	0.53	0.97	0.28	29.85
Bacterial necromass carbon	1.93	0.38	1.00	0.33	25.9

Figure S1. Grazing experimental design and site location total area of the grazing pasture is 52.8 ha with individual plot size of 4.4 ha. NG: No grazing (no sheep per grazing plot), LG: light grazing (4 sheep per grazing plot), MG: moderate grazing (8 sheep per grazing plot, and HG: heavy grazing (12 sheep per grazing plot).

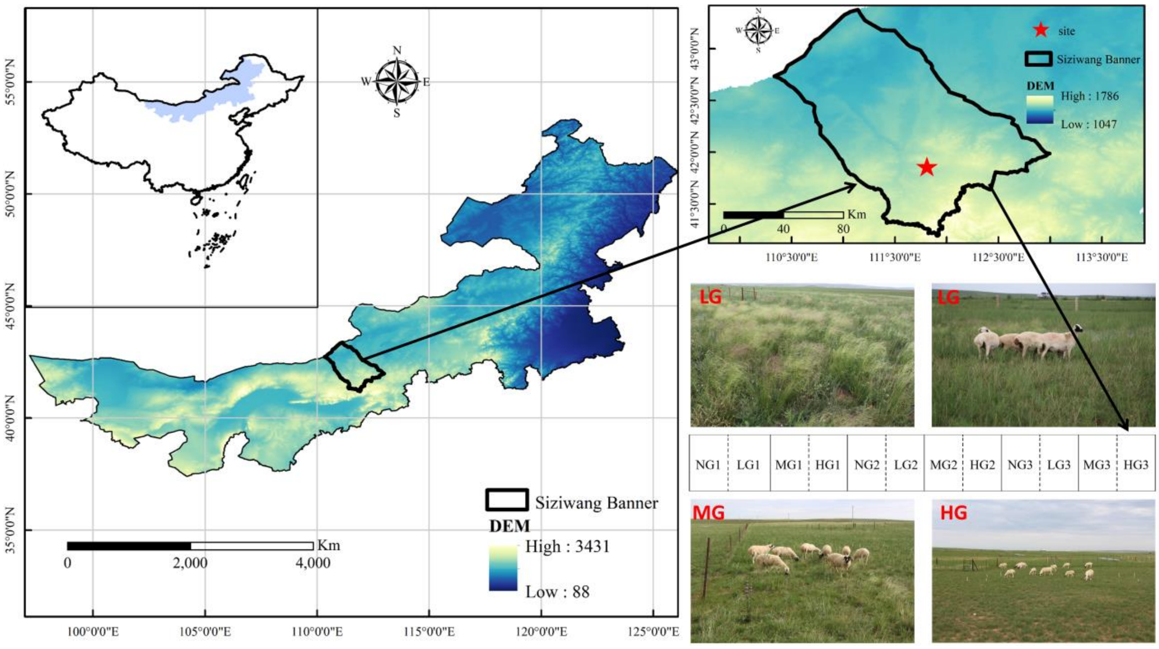

Figure S2. Initial structural equation model considering all plausible pathways through which experimental treatments could influence fungal and bacterial necromass C.

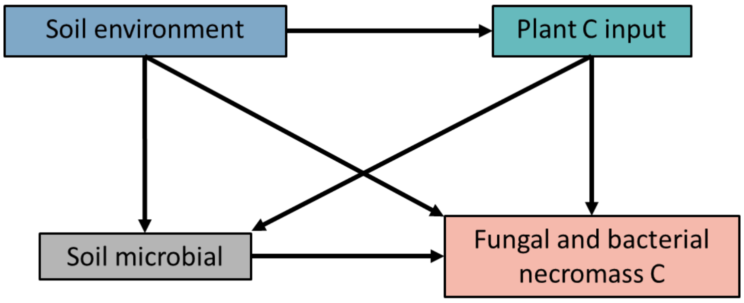

Figure S3. Correlation between relative abundance of soil microorganisms, enzyme activity, plant carbon input and carbon fraction. SOC: Soil organic carbon(g·kg^{-1}); FNC: Fungal necromass carbon(g·kg^{-1}); BNC: Bacteria necromass carbon(g·kg^{-1}); MNC: Microbial necromass carbon(g·kg^{-1}); MBC: Microbial biomass carbon(mg·kg^{-1}); αGC: α-1,4-glucosidase(nmol·g^{-1}·h^{-1}); βGC: β-1,4-glucosidase(nmol·g^{-1}·h^{-1}); NGC: β-1,4-N-acetylglucosaminidase(nmol·g^{-1}·h^{-1}); plant carbon input(g·C·m^{-2}), LA, SA and MA represent the content of large aggregates, small aggregates and microaggregates, respectively. "*" represents a significant correlation between two indicators. The significance level is $P < 0.05$. The shade of the color represents the strength of the correlation.

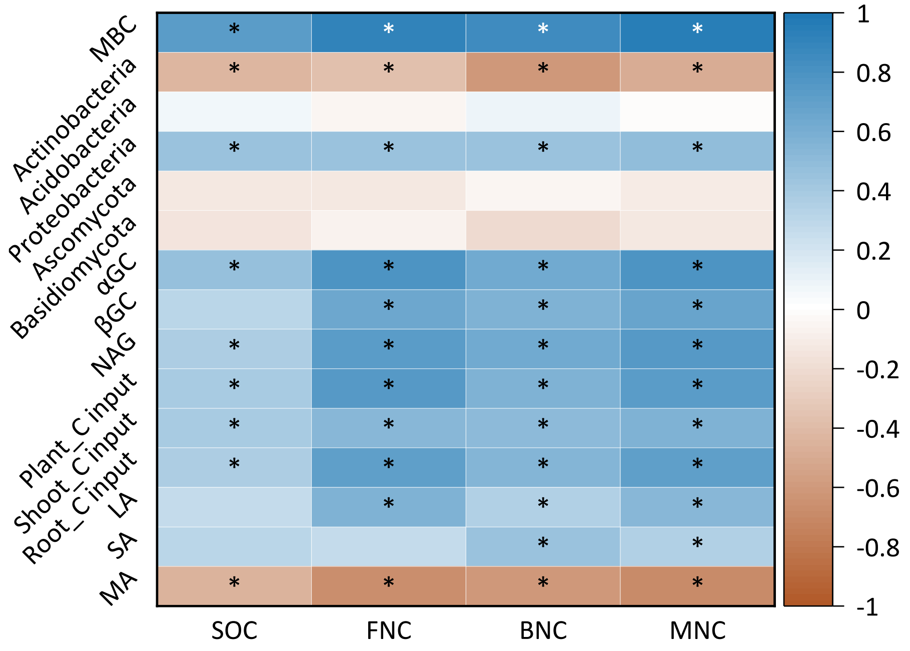

Supplement: Supplementary file 1 [file Data_Sheet_1.pdf]
